# Supplementary material for: WordCommentsAnalyzer: A windows software tool for qualitative research
Source: F1000Res. 2018 Sep 4;7:536. Originally published 2018 May 3. [Version 2] doi: 10.12688/f1000research.14819.2 (PMC6137411; doi:10.12688/f1000research.14819.2)
Supplement: Supplementary file 3 [file f1000research-7-17614-s0002.tgz › b2a79496-4ada-47c4-9682-a99ce867836d.zip!/Eight Iranian Health Journals/Archives of Rehabilitation_8.docx]

© Archives of Rehabilitation. This work is licensed under CC BY-NC 4.0 (https://creativecommons.org/licenses/by-nc/4.0/).

Volume & Issue: دوره 17 - شماره 2

Title: رشد جنبه معنایی فعل در کودک فارسی‌زبان: مطالعه طولی

http://rehabilitationj.uswr.ac.ir/article-1-1593-fa.html

هدف فراگیری فعل به‌عنوان یکی از اجزای اصلی جمله همواره به‌عنوان یکی از مقوله‌های سؤال‌برانگیز در روند فراگیری زبان محسوب شده است. در این میان یکی از مسائل مهم در فعل‌آموزی، تشخیص معنای فعل با استفاده از سرنخ‌های نحوی و فراگیری جنبه معنایی فعل است. بنابراین هدف اصلی پژوهش حاضر بررسی رشد جنبه معنایی فعل در یک کودک فارسی‌زبان بود. علاوه‌براین همان‌گونه که در برخی از زبان‌ها گزارش شده است، پاسخ به این پرسش‌ها بود: آیا در فرایند فراگیری فعل و تعبیر معنای فعلِ کودک فارسی‌زبان، سرنخ‌های نحوی نقشی اساسی ایفا می‌کند؟ آیا کودک در تعبیر معنای فعل از قالب نحوی جمله کمک می‌گیرد؟  روش بررسی این پژوهش به‌صورت موردی و طولی روی یک کودک فارسی‌زبان بین سنین 21 تا 45 ماهگی انجام و اطلاعات به‌طورطبیعی و در بافت مکالمات روزمره جمع‌آوری شد. پس از گردآوری داده‌ها که در طول بیست‌وچهار ماه انجام گرفت، افعال تولید‌شده براساس طبقه‌بندی معنایی افعال در چارچوب دیدگاه لی و نایگلس (2005) در چهار طبقه معنایی افعال حرکتی، افعال درونی-ارتباطی، افعال فرایندهای بدنی و افعال خلقت-اجرایی دسته‌بندی شد. چارچوب نظری پژوهش راه‌اندازی نحوی نایگلس (1990 و 2005) بود که دیدگاهی تعاملی (معنایی-نحوی) محسوب می‌شد. همچنین روش تحلیل داده‌های پژوهش نیز از نوع توصیفی-تحلیلی بود. یافته‌ها یافته‌های حاصل از پژوهش حضور راه‌اندازی نحوی را در روند رشد فعل‌آموزی کودک فارسی‌زبان تأیید می‌کند؛ به این ترتیب که کودک فارسی‌زبان در دوره سنی 21 تا 45 ماهگی از سرنخ‌های نحوی در تعبیر معنای فعل استفاده می‌کند و قالب نحوی جمله به کودک کمک می‌کند که معنای فعل را تعبیر کند. از دیگر دستاوردهای این پژوهش، نشان‌دادن سلسله‌مراتب ظهور طبقه معنایی افعال در روند زبان‌آموزی بود؛ به این صورت که شواهد حاصل از پژوهش نشان داد که در روند فعل‌آموزی کودک فارسی‌زبان، ابتدا طبقه فعل‌های حرکتی ظاهر می‌شود و از بیشترین بسامد رخداد در روند فراگیری فعل برخوردار است. پس‌ازآن فعل‌های فرایندهای بدنی و سپس فعل‌های خلقت-اجرایی و افعال درونی-ارتباطی با بسامد کمتر در این بازه سنی تولید می‌شود. بسامد کم افعال درونی-ارتباطی و نیز درک‌نکردن معنای ضمنی برخی افعال توسط کودک، به‌ویژه در دوره‌های ابتدایی این بازه سنی فعل‌آموزی، نشان می‌دهد که در آغاز بیشتر پدیده‌های ملموس و غیرانتزاعی در ذهن کودک وجود دارد. به‌عبارت‌دیگر کودک پدیده‌های ملموس و غیرانتزاعی را سریع‌تر یاد می‌گیرد و به‌تدریج با یادگیری واژه‌های ملموس و عینی، ذهن نقشی کودک خودبه‌خود به‌دنبال ویژ‌گی‌های مشترک بین واژه‌های ملموس و انتزاعی می‌گردد و آنها را درمی‌یابد. یکی از مسائل قابل‌توجه در یافته‌های پژوهش حاضر این بود که هرچه تعامل کودک با محیط اجتماعی بیشتر می‌شود و رشد شناختی کودک افزایش می‌یابد، تولید فعل به‌ویژه فعل‌های خلقت-اجرایی در کودک افزایش می‌یابد. نتیجه‌گیری شواهد به‌دست‌آمده از پژوهش نشان داد که بسامد روند رشد فعل به‌لحاظ معنایی در کودک فارسی‌زبان مانند کودکان دیگر زبان‌هاست و قالب نحوی جمله در تعبیر معنای فعل به او کمک می‌کند. به‌نظر می‌رسد از این ویژگی می‌توان در تشخیص، ارزیابی و توانبخشی اختلالات گفتار و زبان در روند فراگیری فعل بهره گرفت.

Title: رابطه استرس والد-کودک با مهارت‌های رشدی و تحولی کودکان کاشت حلزون‌شده

http://rehabilitationj.uswr.ac.ir/article-1-1766-fa.html

هدف یکی از درمان‌هایی که اخیراً برای کودکان ناشنوا مطرح شده، کاشت حلزون شنوایی است. کاشت حلزون فناوری جدیدی در تجهیزات شنوایی و روش درمانی پذیرفته‌شده‌ای برای کودکان کم‌شنوا محسوب می‌شود. در سال‌های اخیر پژوهشگران به نقش خانواده و رابطه آن با کسب مهارت‌های رشدی در کودکان مبتلا به آسیب شنوایی توجه ویژه مبذول داشته‌اند. تجربیات بالینی نیز نشان داده است که عملکرد عاطفی و مشکلات روان‌شناختی مراقب اصلی، اثر نامطلوبی بر مهارت‌های رشدی و تحولی کودکان کاشت حلزون‌شده خواهد داشت. پژوهش حاضر با هدف تعیین رابطه استرس والد-کودک با مهارت‌های رشدی و تحولی کودکان کاشت حلزون‌شده انجام شده‌است.  روش بررسی پژوهش حاضر مطالعه‌ای پیمایشی از نوع همبستگی بود که در سال 1393 انجام شد. جامعه آماری این تحقیق را 268 نفر از کودکانی تشکیل می‌دادند که در بیمارستان الزهرای اصفهان عمل جراحی کاشت حلزون روی آنها انجام شده بود. از این جامعه 57 کودک (34 پسر و 23 دختر) که حداقل دو سال از عمل جراحی آنها گذشته بود، به روش تصادفی انتخاب و بررسی شدند. برای سنجش متغیرهای پژوهش از شاخص تنیدگی والدینی، مقیاس عملکرد بین‌المللی لایتر، مقیاس رشد اجتماعی وایلند، مقیاس رشد و تحول زبان و مقیاس عملکرد ارتباطی کودکان کاشت حلزون‌شده استفاده شد. داده‌های پژوهش با استفاده از نسخه 22 نرم‌افزار SPSS آمار توصیفی، روش همبستگی و تحلیل رگرسیون تجزیه‌وتحلیل شد. یافته‌ها نتایج حاصل از تحلیل داده‌ها نشان داد بین استرس والد-کودک با رشد زبانی (59/0-)، اجتماعی (38/0-) و ارتباطی (54/0-)کودک رابطه منفی (معکوس) معنی‌داری وجود دارد (01/0P≥). با توجه به نتایج تحلیل رگرسیون در تبیین رشد زبانی (متغیر ملاک) کودک براساس استرس والد-کودک (متغیر پیش‌بین) ملاحظه می‌شود که ضریب تبیین برابر 34/0=R2 است و نتایج به‌دست‌آمده نشان می‌دهد که 34 درصد واریانس متغیر وابسته (رشد زبانی) از طریق استرس والد-کودک تعیین می‌شود. همچنین در تبیین رشد اجتماعی کودک (متغیر پیش‌بین) براساس استرس والد-کودک (متغیر ملاک) ملاحظه می‌شود که ضریب تبیین برابر 14/0=R2 است و نتایج به‌دست‌آمده نشان می‌دهد 14 درصد واریانس متغیر وابسته (رشد اجتماعی) از طریق استرس والد-کودک تعیین می‌شود. علاوه‌براین در تبیین عملکرد ارتباطی کودک (متغیر پیش‌بین) براساس استرس والد-کودک (متغیر ملاک) مشاهده می‌شود که ضریب تبیین برابر 29/0=R2 است و نتایج به‌دست‌آمده از این امر حکایت دارد که 29 درصد واریانس متغیر وابسته (رشد اجتماعی) از طریق استرس والد-کودک تعیین می‌شود. نتیجه‌گیری درمجموع یافته‌های این پژوهش نشان داد که استرس والد-کودک، بر بخش قابل‌توجهی از مهارت‌‌های رشدی و تحولی کودکان کاشت حلزون‌شده تأثیر می‌گذارد. با توجه به اهمیت و ضرورت رشدی و تحولی در کودکان کاشت حلزون‌شده، نتایج این پژوهش ضرورت ارائه خدمات مشاوره‌ای و حمایت‌های روانشناختی را برای این گروه از والدین گوشزد می‌کند. براین‌اساس می‌توان برنامه‌هایی را در قالب کارگاه‌های آموزشی برای مادران این کودکان پیشنهاد کرد. همچنین به روانشناسان و مشاوران خانواده و متخصصان خانواده درمانی توصیه می‌شود که در برنامه‌های درمانی و آموزشی خود به نقش فشار روانی در روابط والد-کودک توجه ویژه‌ای داشته باشند.

Title: مقایسه حس عمقی مفصل شانه در زنان با و بدون‌شلی عمومی مفاصل

http://rehabilitationj.uswr.ac.ir/article-1-1794-fa.html

هدف شلی عمومی مفاصل افراد را مستعد ابتلا به انواع ضایعات عضلانی-اسکلتی به‌ویژه در مفصل شانه می‌کند. حس عمقی که شامل حس وضعیت و حس حرکت مفصل است، نقش مهمی در ثبات عملکردی شانه دارد. با توجه به نقش مهم حس عمقی در عملکرد مفصل شانه این پژوهش با هدف انجام انجام شد: 1. مقایسه حس وضعیت و حس حرکت مفصل شانه در افراد با و بدون‌شلی عمومی مفاصل؛ 2. مقایسه حس عمقی بین دو سمت غالب و غیرغالب در هر گروه. روش بررسی در این مطالعه موردی-شاهدی 20 زن با شلی عمومی مفاصل (سن30/20±05/22 سال) و 20 زن بدون‌شلی مفصلی عمومی (سن53/2±65/22 سال) شرکت کردند. آزمون‌ها در وضعیت طاق‌باز انجام شد. قبل از شروع هر آزمون افراد با مراحل انجام آزمون‌ها آشنا می‌شدند. حس عمقی شامل آزمون بازسازی زاویه‌ای غیرفعال و حس حرکت بود که با دستگاه دینامومتر ایزوکینتیک انجام شد. ابتدا دامنه حرکتی چرخش خارجی شانه با گونیومتر استاندارد اندازه‌گیری و 90 درصد انتهایی آن به‌عنوان زاویه هدف در آزمون بازسازی زاویه‌ای غیرفعال درنظرگرفته شد. سپس حس حرکت نیز به روش آستانه تشخیص حرکت غیرفعال در صفر درجه چرخش خارجی اندازه‌گیری شد. آزمون آستانه تشخیص حرکت با سرعت 5/0 درجه بر ثانیه و آزمون بازسازی زاویه‌ای با سرعت 2 درجه بر ثانیه انجام گرفت. آزمون‌ها در هر دو شانه غالب و غیرغالب انجام شد. توالی انجام آزمون‌ها برای آزمون بازسازی زاویه‌ای و آستانه تشخیص حرکت و نیز شانه غالب و غیرغالب به‌صورت تصادفی بوده است. برای حذف بازخوردهای بینایی و شنوایی از چشم‌بند و گوشی استفاده شد. میانگین خطای بازسازی زاویه غیرفعال و میانگین آستانه تشخیص حرکت غیرفعال در سه بار تکرار آزمون ثبت شد. از آزمون تی مستقل برای مقایسه حس بازسازی زاویه‌ای غیرفعال و حس حرکت بین دو گروه و آزمون تی زوج برای مقایسه دو سمت غالب و غیرغالب در هرکدام از گروه‌ها استفاده شد. یافته‌ها تفاوت معناداری در آستانه تشخیص حرکت در سمت غالب و غیرغالب بین دو گروه مشاهده نشد [در شانه غالب (47/0=P) و در شانه غیرغالب (70/0=P)]. در سمت غالب و غیرغالب، زنان با شلی عمومی مفاصل خطای بیشتری در بازسازی زاویه غیرفعال حرکت چرخش خارجی شانه در مقایسه با زنان بدون‌شلی مفصلی عمومی نشان دادند (001/0(P=. تفاوت معنی‌داری بین حس وضعیت و حرکت بین دو سمت غالب و غیرغالب در هرکدام از گروه‌ها مشاهده نشد (P>0/05). نتیجه‌گیری نتایج تحقیق نشان داد حس بازسازی زاویه در دامنه انتهایی شانه در افراد مبتلا به شلی عمومی مفاصل در مقایسه با افراد بدون‌شلی عمومی مفاصل کاهش می‌یابد. این امر می‌تواند حاصل اختلال یا کاهش پیام‌های حسی گیرنده‌های مفصلی وارده به سیستم عصبی مرکزی باشد. کاهش بازخورد حس عمقی ممکن است به این منجر شود که اندام از نظر بیومکانیکال وضعیت نادرستی را اتخاذ کند. چنین سازوکاری می‌تواند به تغییرات تخریبی مفصل سرعت بخشد و دلیلی برای شیوع بالای مشکلات عضلانی اسکلتی در افراد با شلی عمومی مفصلی باشد.

Title: تأثیر غوطه‌وری ساق و مچ پا در آب سرد بر تعادل پویای ورزشکاران و غیرورزشکاران

http://rehabilitationj.uswr.ac.ir/article-1-1820-fa.html

هدف در سال‌های اخیر غوطه‌وری در آب سرد (CWI) پس از جلسات تمرینی سنگین و مسابقات، با هدف تسریع زمان احیای ورزشکاران رواج یافته است. در چندین مطالعه اثر غوطه‌وری در آب سرد را بر عملکرد و عوامل آمادگی جسمانی ورزشکاران بررسی شده است؛ اما در این میان در پژوهش‌های اندکی تأثیر غوطه‌وری در آب سرد را بر تعادل پویای ورزشکاران و غیرورزشکاران بررسی شده است. بنابراین هدف تحقیق حاضر بررسی تأثیر هشت دقیقه سرمادهی موضعی مچ پا و پا به‌وسیله غوطه‌وری در آب سرد بر شاخص‌های تعادل پویای مردان و زنان ورزشکار و غیرورزشکار سالم بوده است.  روش بررسی در این پژوهش 58 نفر از دانشجویان دانشگاه تهران (29 نفر ورزشکار و 29 نفر غیرورزشکار) با سن 2±2/22 سال، وزن 2±‌2/69 کیلوگرم و قد 2±2/175 سانتی‌متر داوطلبانه شرکت کردند و به دو گروه ورزشکار و غیر ورزشکار تقسیم شدند. شاخص‌های تعادل کلی، قدامی-خلفی و طرفی آزمودنی‌ها به‌وسیله سیستم ثباتی بایودکس (BSS) مدل SW45-30D-E617 ساخت کشور آمریکا ارزیابی شد. به‌منظور آشنایی آزمودنی‌ها با سیستم بایودکس و کسب آمادگی برای ارزیابی تعادل پویا، هر آزمودنی به‌مدت یک دقیقه روی دستگاه تمرین می‌کرد. سپس هر آزمودنی به‌مدت هشت دقیقه هر دو پای خود را درون ظرف آب و یخ صفر درجه قرار می‌داد؛ به‌نحوی‌که کف پا و تمام اطراف پا تا پنج سانتی‌متر بالای ناحیه قوزک تحت‌تأثیر سرما قرار می‌گرفت. آزمون تعادل بلافاصله پس از بیرون‌آمدن ورزشکار از ظرف مخلوط یخ و آب، باردیگر روی دستگاه بایودکس تکرار شد. یافته‌های تحقیق با استفاده از آزمون آماری آنوا در سطح 05/0>P تجزیه‌وتحلیل شد.  یافته‌ها نتایج تحقیق نشان داد که اختلاف معنی‌داری در شاخص ثبات کلی قبل (M=2/94 و SD=1/26) و بعد از سرمادرمانی (M=2/66  و SD=1/40) وجود ندارد (t(57)=1/29 و P=0/202). همچنین اختلاف معنی‌داری در شاخص ثبات قدامی خلفی قبل (M=2/27 وSD=1/06) و بعد از سرمادرمانی (M=2/08 و SD=1/19) مشاهده نشد (t(57)=0/98 و P=0/33). باوجوداین شاخص ثبات طرفی بعد از سرمادرمانی (M=1/78 و SD=0/88) به‌طور معنی‌داری در مقایسه با قبل از اعمال سرمادرمانی (M=2/05 و SD=0/84) افزایش یافت (t(57)=2/10 و P=0/03). همچنین نتایج آزمون تحلیل واریانس با اندازه‌گیری‌های مکرر نشان داد که اثر ورزشکاربودن یا نبودن افراد بر شاخص ثبات کلی و قدامی-خلفی (84/0=(P و شاخص ثبات طرفی (18/0=(P قبل و بعد از اعمال سرما معنی‌دار نیست. نتیجه‌گیری نتایج تحقیق حاضر نشان داد که اعمال سرما از طریق غوطه‌وری در آب سرد به‌مدت هشت دقیقه بر شاخص ثبات کلی و قدامی خلفی در مردان و زنان ورزشکار و غیرورزشکار اثر معنی‌داری ندارد. به‌نظر می‌رسد که سرمای به‌کاررفته در این تحقیق تنها بر گیرنده‌های پوستی تأثیر گذاشته و بر گیرنده‌های مفصلی عمقی‌تر که نقش اصلی در تعادل دارند، ‌تأثیر نگذاشته است.

Title: بررسی تأثیر دو نوع راکر کفش بر متغیرهای زمانی-مکانی راه‌رفتن در بیماران دیابتی

http://rehabilitationj.uswr.ac.ir/article-1-1849-fa.html

هدف زخم کف پا از عوارض مهم بیماران دیابتی است که بر توانایی ایستادن و راه‌رفتن آنان تأثیر می‌گذارد. راهکارهای درمانی متفاوتی برای کاهش نیروهای اعمالی روی پای این بیماران استفاده شده که اکثراً موقتی است و در بلندمدت مؤثر نیست. کفش‌های راکری برای درمان زخم‌های دیابتی توصیه می‌شود. براساس مقالات موجود این سؤال مطرح می‌شود: «آیا نیروهای اعمالی روی پا در افراد دیابتی با افراد سالم متفاوت است؟» علاوه‌براین مشخص نیست کدام نوع راکر (پنجه یا پاشنه) برای کاهش این نیروها مؤثرتر است؛ بنابراین هدف از این مطالعه مقایسه بین نیروهای اعمالی وارد بر پا در افراد دیابتی و افراد سالم و همچنین تعیین تأثیر راکرها بر این عوامل بود. فرضیه اصلی مطرح‌شده در این مطالعه بر این مبنا بود که راکرهای پنجه و پاشنه تأثیر مشابهی بر نیروهای اعمالی وارد بر پا در افراد دیابتی دارد. روش بررسی در این مطالعه مداخله‌ای‌شبه‌تجربی 20 نفر فرد سالم و 20 نفر فرد بیمار مبتلا به نروپاتی دیابتی شرکت داشتند. تعداد نمونه‌ها براساس متوسط تعداد شرکت‌کنندگان در مطالعات قبلی انتخاب شده بودند. نمونه‌ها از میان بیماران ارجاع‌داده‌شده به کلینیک مراقبت از پای دانشکده توانبخشی دانشگاه علوم پزشکی اصفهان انتخاب شدند. متغیرهای زمانی-مکانی راه‌رفتن و نیروهای اعمالی روی پا طی راه‌رفتن با استفاده از سیستم تحلیل حرکت کوالیزیز مجهز به 7 دوربین و یک صفحه نیروی کیسلر (60×50 سانتی‌متر) ثبت شد. از افراد خواسته ‌شد با سرعت دلخواه در طول آزمایشگاه راه بروند تا تأثیر آنی راکرها بر بیماران سنجیده شود. متغیرهایی شبیه متغیرهای زمانی-مکانی راه‌رفتن، حداکثر نیروی عکس‌العمل زمین و انتگرال نیرو-زمان (ضربه) تحلیل شد. تفاوت بین میانگین‌ها و تأثیرات بین افراد با استفاده از آزمون تحلیل واریانس با اندازه‌گیری مکرر ارزیابی و محاسبات آماری با استفاده از نسخه 17 نرم‌افزار SPSS و با سطح معنی‌داری 05/0 انجام شد. یافته‌ها براساس نتایج به‌دست‌آمده اختلاف عمده‌ای بین افراد دیابتی و سالم در متغیرها آهنگ راه‌رفتن، طول گام و درصد مرحله استانس مشاهده می‌شود (05/0>P). با وجود اینکه سرعت راه‌رفتن افراد دیابتی کمتر از افراد سالم بود؛ ولی اختلاف معناداری در این متغیر مشاهده نشد (06/0P). به نظر می‌رسد متوسط انتگرال نیرو-زمان بخش عمودی نیروی عکس‌العمل زمین براساس متغیر سمت و گروه متفاوت است (05/0>P). نتیجه‌گیری انتگرال نیرو-زمان بخش عمودی نیروی عکس‌العمل زمین به‌طورعمده در افراد دیابتی بیشتر از افراد سالم بود. این امر می‌تواند زخم‌هایی در پای این افراد ایجاد کند. البته راکرهای پاشنه و پنجه در کاهش هیچ‌کدام از متغیرهای میزان نیروهای اعمالی و انتگرال نیرو-زمان مؤثر نبودند. توصیه می‌شود برای افزایش ثبات پویا و کاهش فشارهای کف‌پایی از کفش‌های راکری با سطح تکیه‌گاه عریض‌تر استفاده شود. مهم‌ترین محدودیت این مطالعه بررسی تأثیر آنی استفاده از راکر در این افراد بود. در مطالعات بعدی پیشنهاد می‌شود که تأثیر مداخلات راکری بعد از یک دوره استفاده ارزیابی شود.

Title: تأثیر هشت هفته بازی‌های اصلاحی بر میزان انحنای کایفوز و قدرت عضلات تنه کودکان کم‌توان ذهنی

http://rehabilitationj.uswr.ac.ir/article-1-1671-fa.html

هدف در جامعه ما گروه وسیعی از کودکان و بزرگسالان هستند که به‌گونه‌ای خاص از نظر جنبه‌های فکری، جسمی، عاطفی یا ویژگی‌های رفتاری با دیگر همسالان خود تفاوت دارند. یکی از این اقشار آسیب‌پذیر کودکان کم‌توان ذهنی هستند که به‌علت شرایط ذهنی و روانی ویژه درمقایسه با افراد سالم تحرک کمتری دارند و به‌همین علت به ضعف‌های جسمانی-حرکتی دچار می‌شوند. براین‌اساس با توجه به نیاز این کودکان به بازی برای ایجاد شادابی و نبود پژوهش‌هایی در زمینه حرکات اصلاحی در قالب بازی، هدف از انجام این پژوهش تأثیر هشت هفته بازی‌های اصلاحی بر میزان انحنای کایفوز و قدرت عضلات تنه کودکان کم‌توان ذهنی بود.  روش بررسی برای انجام این تحقیق 30 کودک کم‌توان ذهنی مبتلا به ناهنجاری کایفوز با زاویه کایفوز بیشتر از 40 درجه به‌طورتصادفی به دو گروه 15 نفره تجربی با میانگین سنی 8/2±06/12 سال،  قد 1/0±4/1 متر و وزن 38/1±3/42 کیلوگرم و‌ گروه کنترل با میانگین سنی 8/2±2/12 سال، قد 9/0±4/1 متر و وزن 3/1±6/43 کیلوگرم تقسیم شدند. هر دو گروه از نظر ویژگی‌های آنتروپومتریک باهم همسان بودند. برای اندازه‌گیری زاویه کایفوز از خط‌کش منعطف و برای بررسی قدرت عضلات بازکننده ستون فقرات از دستگاه دینامومتر استفاده شد. گروه تجربی طی یک دوره هشت هفته‌ای (سه جلسه در هفته) بازی‌های اصلاحی منتخب را انجام دادند. در این مدت گروه کنترل هیچ‌گونه تمرینی را تجربه نکردند. پس از پایان تمرینات، میزان انحنای کایفوز و قدرت عضلات بازکننده تنه هر دو گروه باردیگر ارزیابی شد. به‌منظور تجزیه‌وتحلیل داده‌ها از نسخه 16 نرم‌افزار SPSS و برای بررسی اختلاف بین دو گروه از آزمون تی مستقل در سطح 05/0 استفاده شد (05/0P≤). یافته‌ها نتایج حاصل از این پژوهش نشان داد که بین دو گروه تجربی و کنترل در میزان انحنای کایفوز (t=4/41 و P=0/001) و قدرت عضلات بازکننده تنه (t=3/15 و P=0/004) بعد از هشت هفته تمرینات اصلاحی منتخب اختلاف معناداری وجود داشت و گروه تجربی بهبودیِ معناداری داشتند. نتیجه‌گیری با توجه به نتایج تحقیق حاضر مبنی‌بر تأثیر بازی‌های اصلاحی بر میزان انحنای کایفوز و قدرت عضلات تنه کودکان کم‌توان ذهنی و نیاز این کودکان به حفظ زندگی مستقل و رفع ناهنجاری‌های جسمانی پیشنهاد می‌شود پروتکل‌های بازی‌درمانی به‌منظور رفع ناهنجاری‌های جسمانی این کودکان انجام شود.
